# Early exercise induces long-lasting morphological changes in cortical and hippocampal neurons throughout of a sedentary period of rats

**DOI:** 10.1038/s41598-019-50218-9

**Published:** 2019-09-23

**Authors:** Fernando Tadeu Serra, Andrea Dominguez Carvalho, Bruno Henrique Silva Araujo, Laila Brito Torres, Fabrizio dos Santos Cardoso, Jéssica Salles Henrique, Eduardo Varejão Díaz Placencia, Roberto Lent, Fernando Gomez-Pinilla, Ricardo Mario Arida, Sérgio Gomes da Silva

**Affiliations:** 1 Research and Technology Center, University of Mogi das Cruzes, Mogi das Cruzes, SP Brazil; 2 Federal University of São Paulo (UNIFESP), São Paulo, SP Brazil; 3 Brazilian Biosciences National Laboratory (LNBio) Brazilian Center for Research in Energy and Materials (CNPEM), Campinas, SP 13083-970 Brazil; 4 Department of Genetics and Evolutionary Biology, Human Genome and Stem Cell Research Center, Biosciences Institute, University of São Paulo (USP), São Paulo, SP Brazil; 5 Faculdade São Leopoldo Mandic, Área de Fisiologia, Farmacologia, Instituto São Leopoldo Mandic, Campinas, Brazil; 6 Institute of Biomedical Sciences, Federal University of Rio de Janeiro (UFRJ), Rio de Janeiro, RJ Brazil; 7 D’Or Institute for Research and Education (IDOR), Rio de Janeiro, RJ Brazil; 8 University of California Los Angeles (UCLA), Los Angeles, CA USA; 9 Hospital Israelita Albert Einstein (HIAE), São Paulo, SP Brazil; 10 Centro Universitário FAMINAS (UNIFAMINAS), Muriaé, MG Brazil; 11 Hospital de Câncer de Muriaé, Fundação Cristiano Varella (FCV), Muriaé, MG Brazil

**Keywords:** Neural ageing, Neurotrophic factors

## Abstract

Life experiences at early ages, such as physical activity in childhood and adolescence, can result in long-lasting brain effects able to reduce future risk of brain disorders and to enhance lifelong brain functions. However, how early physical exercise promotes these effects remains unclear. A possible hypothesis is that physical exercise increases the expression of neurotrophic factors and stimulates neuronal growth, resulting in a neural reserve to be used at later ages. Basing our study on this hypothesis, we evaluated the absolute number and morphology of neuronal cells, as well as the expression of growth, proliferation and survival proteins (BDNF, Akt, mTOR, p70S6K, ERK and CREB) in the cerebral cortex and hippocampal formation throughout of a sedentary period of rats who were physically active during youth. To do this, male Wistar rats were submitted to an aerobic exercise protocol from the 21^st^ to the 60^th^ postnatal days (P21–P60), and evaluated at 0 (P60), 30 (P90) and 60 (P120) days after the last exercise session. Results showed that juvenile exercise increased, and maintained elevated, the number of cortical and hippocampal neuronal cells and dendritic arborization, when evaluated at the above post-exercise ages. Hippocampal BDNF levels and cortical mTOR expression were found to be increased at P60, but were restored to control levels at P90 and P120. Overall, these findings indicate that, despite the short-term effects on growth and survival proteins, early exercise induces long-lasting morphological changes in cortical and hippocampal neurons even during a sedentary period of rats.

## Introduction

There is a famous quote from Oliver Wendell Holmes that says: “*Man*’*s mind, once stretched by a new idea, never regains its original dimensions*”. These words have special meaning because they reflect the importance of life experiences. In this context, it has been reported that early life experiences, such as physical activity in childhood and adolescence, can induce long-lasting brain effects^1–3^. For instance, a prospective population-based study showed a positive relationship between physical activity at 15 and 25 years of age and information processing speed at 62 and 85 years of age^2^. In another study, physical fitness at age 18 predicted occupational status and educational achievement later in life^1^. It has been also observed that older women who were physically active during their adolescence showed better cognitive performance and had a lower likelihood of cognitive impairment in late life compared with those who were physically inactive^3^. These interesting data support the hypothesis that being physical active in early life can result in a “neural reserve” throughout the lifespan that may enhance lifelong brain functions and reduce the future risk of brain disorders^4^. However, how early physical activity promotes these effects remains unclear.

Data from basic research in animal models has indicated that juvenile physical exercise can influence the brain’s functional integrity in adulthood through neuroplastic processes^5^. Studies on rodents have shown that early exercise increases axonal and neuronal density and enhances brain-derived neurotrophic factor (BDNF) expression and its receptor tropomyosin-related kinase B (TrkB) in the hippocampal formation^6,7^, a brain structure which is related to mnemonic and emotional processes. Concomitantly, improved learning and memory in adulthood have been observed in rats exposed to exercise when they were young^6,7^. Another important finding is that early physical activity improves the ability to evoke spatial memories in later life^7^. This finding supports previous research in humans that has shown a correlation between physical activity at an early age and long-lasting benefits on brain functions^2^.

Taken together, these findings indicate that early physical exercise may enable better neural development. In support of this view, a significant cellular proliferative effect of aerobic exercise has been found in the cerebral cortex and hippocampal formation in postnatal development^8^, when the brain is more plastic. Moreover, recent findings have also demonstrated that juvenile exercise produces more robust and sustained health benefits than when practiced in adulthood^9^. Another possible explanation is that early physical activity results in more complex neural circuitry. For instance, a significant increase in long-term potentiation (LTP) has been detected in the dentate gyrus of male adolescent rats trained over fourteen days^10^. It has also been noted that rats physically active in youth exhibit higher neurogenesis and greater dendritic arborization in the dentate gyrus when compared with sedentary rats^11^. Even though these findings are promising, the stability or permanence of brain changes which result from early exercise after it has been interrupted are still unclear.

Therefore, to test whether exercise in early life has long-lasting benefits on the brain, we conducted an experimental study to evaluate the number of neuronal cells and their morphology in the cerebral cortex and hippocampal formation throughout of a sedentary period of rats submitted to physical exercise in adolescence (21^st^ to 60^th^ postnatal day; P21–P60). To do this, we analyzed the absolute number of cells (using the isotropic fractionator method) and the dendritic arborization of neurons (using the Golgi-Cox method). Taking into account the beneficial influence of exercise on growth, proliferation and survival proteins^12–15^, we also evaluated cortical and hippocampal expression of BDNF, kinase B (Akt), mammalian target of rapamycin (mTOR), p70 ribosomal protein S6 kinase (p70S6K), extracellular signal-regulated protein kinase (ERK), and cAMP response element-binding protein (CREB). The neurotrophin BDNF plays an essential role during brain maturation and development^16,17^. When released into the brain, BDNF activates intracellular signaling pathways, such as Akt, mTOR, p70S6K, ERK and CREB, to promote cell proliferation, branching and remodeling of dendrites and axons, as well as functional maturation of excitatory and inhibitory synapses^16,17^. The next step, bearing in mind the positive impact of physical activity for improving cognitive functions and reducing the risk of disease^1–3^, was to analyze these proteins to verify whether early exercise-induced morphological changes are accompanied by lasting alterations in brain expression of proteins linked to cell growth, proliferation and survival (BDNF, Akt, mTOR, p70S6K, ERK and CREB). To evaluate whether early exercise on the treadmill results in different levels of stress, we also measured cortical and hippocampal levels of stress hormones adrenocorticotropic (ACTH) and corticosterone.

## Results

### Absolute number of neuronal and non-neuronal cells

Two-way ANOVA results for the absolute number of neuronal and non-neuronal cells are presented in Supplementary Table 1.

**Figure 1:**
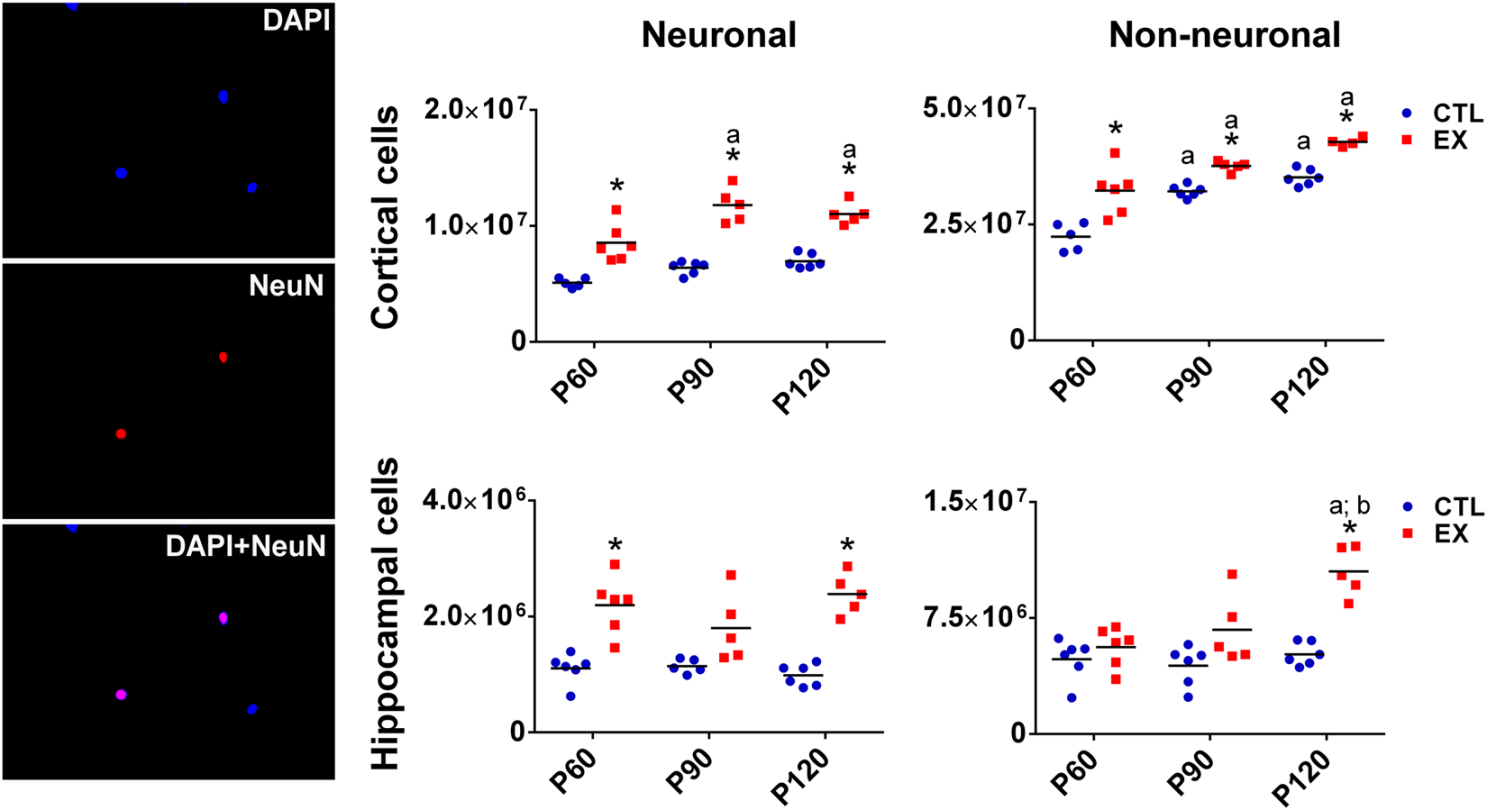
Cortical and hippocampal number of neuronal and non-neuronal cells from the exercise (EX) and control (CTL) groups evaluated using the isotropic fractionator method at 0 (P60), 30 (P90) and 60 (P120) days after the last exercise session (n = 5–6 in each age and group). Representative fluorescence images of neuronal and non-neuronal nuclei stained with 4′-6-diamidino-2-phenylindole dihydrochloride (DAPI) (blue), neuron-specific nuclear protein (NeuN) (red) and DAPI + NeuN (pink in merged image) can be seen on the left. Scale bar = 25 μm. Significant difference compared with P60^a^, P90^b^, P120^c^, or CTL group* (p < 0.05 by two-way ANOVA and Tukey post-test).

Tukey’s post-hoc test showed a significant increase in the number of neurons and non-neuronal cells of the cerebral cortex during the aging of rats from both control (CTL) and exercise (EX) groups, particularly when comparing ages 90 (P90) and 120 (P120) postnatal days with 60 postnatal day (P60) (p < 0.05). In the hippocampal formation, the number of non-neuronal cells was found to be increased at P120 in rats from the EX group (p < 0.05), but not in those from the CTL group (p > 0.05) (Fig. 1).

When comparing studied groups (EX *vs* CTL), post-hoc analysis showed that early exercise increased the cortical number of neuronal and non-neuronal cells at P60, P90 and P120 (p < 0.05). In the hippocampal formation too, more neuronal cells were observed in the EX group compared with the CTL group at P60 and P120 (p < 0.05). An increase in hippocampal non-neuronal cells was only observed in the EX group at P120 (p < 0.05) (Fig. 1).

### Dendritic arborization

Two-way ANOVA results for the number of dendrites, dendritic nodes and ends, and total dendritic length are presented in Supplementary Table 2.

**Figure 2:**
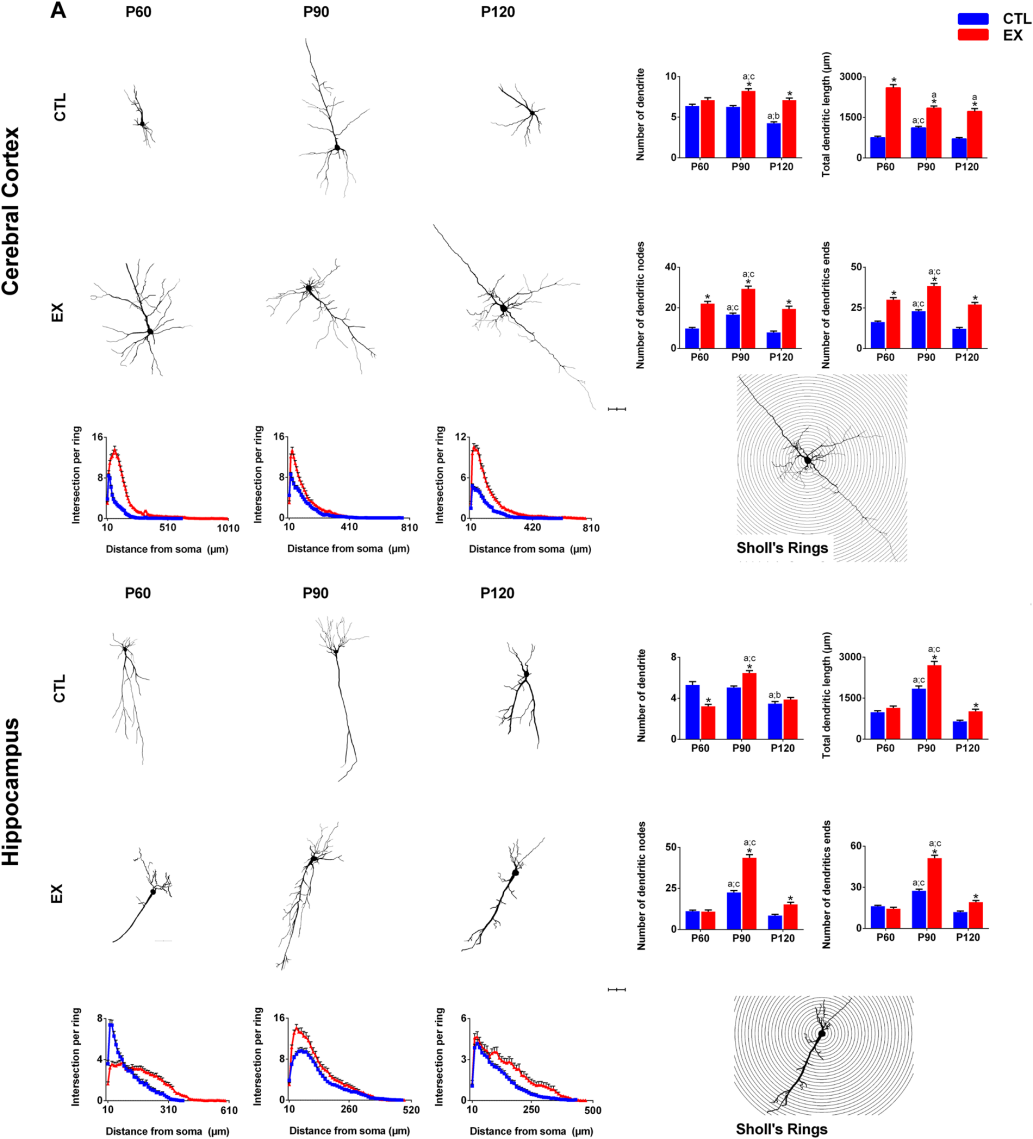
Representative images and quantitative analysis of the dendritic arborization of Golgi-impregnated neurons from the exercise (EX) and control (CTL) groups at 0 (P60), 30 (P90) and 60 (P120) days after the last exercise session (n = 5–6 in each age and group). Number of dendrites, dendritic nodes and ends, and total dendritic length were measured in the cortical neurons of layers and hippocampal neurons of the CA1 subfield (n = 10 neurons/region per rat). Dendritic arborization was calculated by the number of dendrites crossing each circle of 10μm radius around the soma (Sholl’s rings). Scale bar = 50 μm. Significant differences compared with P60^a^, P90^b^, P120^c^, or CTL group* (p < 0.05 by two-way ANOVA and Tukey post-test).

Post-hoc analysis revealed changes in the number of dendrites, total dendritic length, nodes and terminal ends in the cortical neurons located in layers III–V of the motor and somatosensory regions (4–6 neurons per region) and the hippocampal neurons of Cornu Ammonis 1 (CA1) over the age period evaluated, mainly comparing P90 with other ages (p < 0.05) (Fig. 2). When comparing EX and CTL groups, it was observed that, in most cases, early exercise increased dendritic parameters in cortical and hippocampal neurons at P60, P90 and P120 (p < 0.05) (Fig. 2). Sholl analysis conducted on cortical and hippocampal neurons at P60, P90 and P120 also showed more dendritic complexity (intersections on Sholl rings from 20 to 360–550 µm) in exercised rats than in control rats (p < 0.05) (Fig. 2). Taken together, these results show that early physical exercise increased the dendritic arborization of neurons and maintained it at a higher even after a sedentary life period of rats.

### BDNF, ACTH and corticosterone levels

Two-way ANOVA results for cortical and hippocampal levels of BDNF, ACTH and corticosterone are presented in Supplementary Table 3.

**Figure 3:**
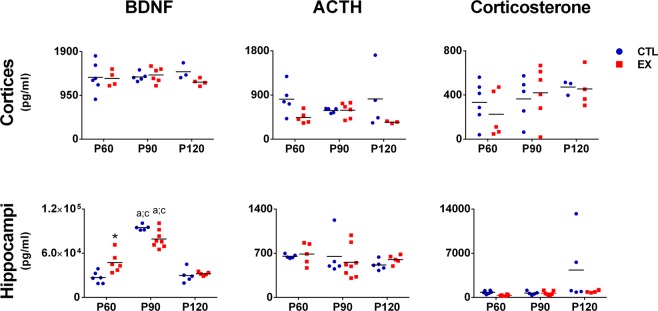
Cortical and hippocampal levels of brain-derived neurotrophic factor (BDNF), adrenocorticotropic (ACTH) and corticosterone hormones from the exercise (EX) and control (CTL) groups at 0 (P60), 30 (P90) and 60 (P120) days after the last exercise session (n = 4–6 in each age and group). Significant difference compared with P60^a^, P90^b^, P120^c^, or CTL group* (p < 0.05 by two-way ANOVA and Tukey post-test).

Post-hoc analysis showed no changes in cortical and hippocampal levels of ACTH or corticosterone over the life course of rats from the EX and CTL groups (p > 0.05), but higher hippocampal BDNF levels were detected in both groups at P90 when compared with P60 and P120 (p > 0.05) (Fig. 3).

Comparing the EX with the CTL group, no significant difference in the ACTH and corticosterone levels was noted (p > 0.05). However, higher levels of hippocampal BDNF were found in rats from the EX group in relation to those from CTL group at P60 (p < 0.05), but this protein was restored to control levels at P90 and P120 (p > 0.05) (Fig. 3).

### Intracellular proteins linked to cell growth, proliferation and survival

Two-way ANOVA results for cortical and hippocampal expression and activation (phosphorylated/total) of Akt, mTOR, p70S6K, ERK and CREB are presented in Supplementary Tables 4 and 5.

**Figure 4:**
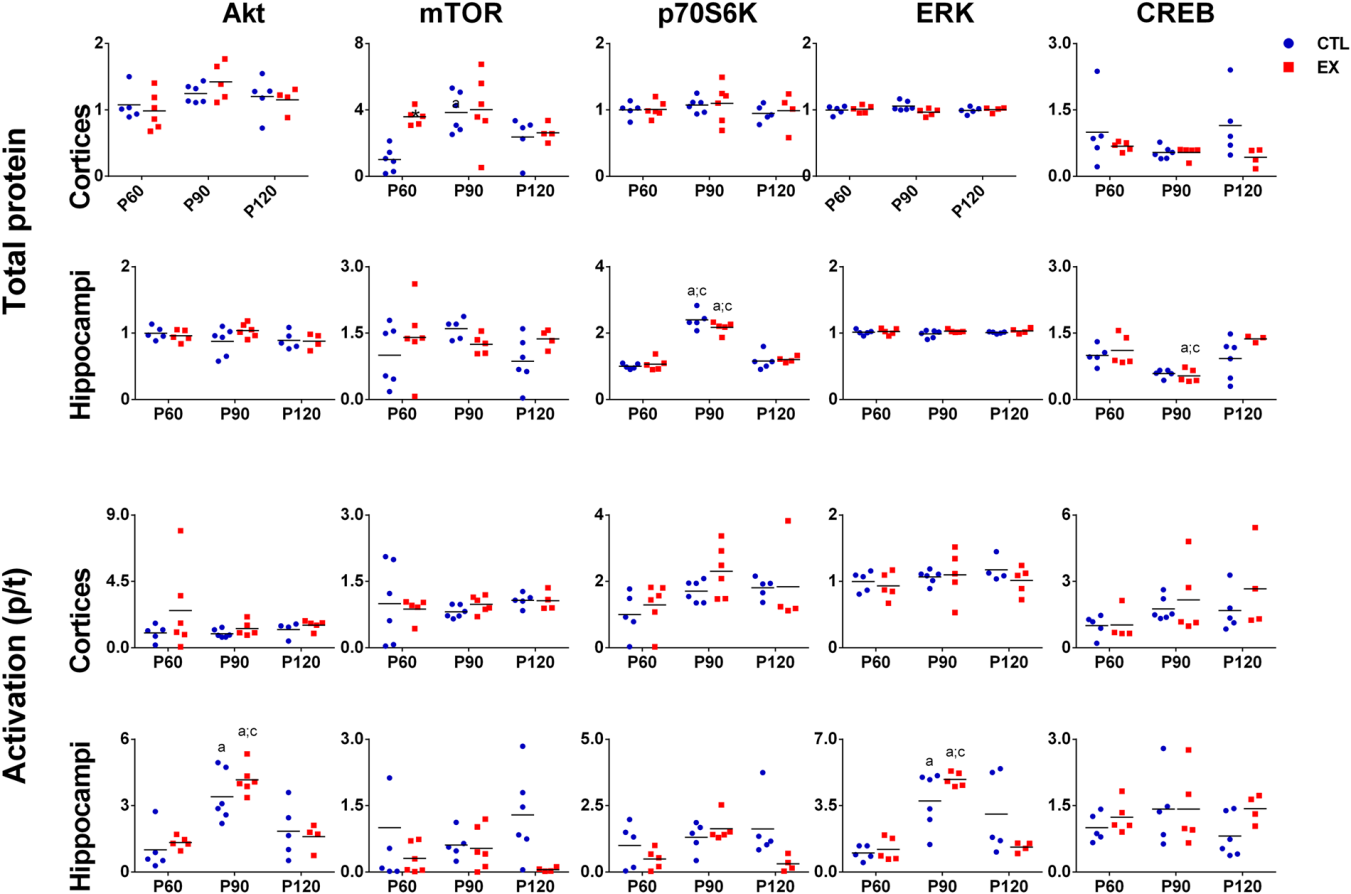
Expression and activation (phosphorylated/total) of Akt, mTOR, p70S6K, ERK and CREB in the cerebral cortex and hippocampal formation of rats from the exercise (EX) and control (CTL) groups at 0 (P60), 30 (P90) and 60 (P120) days after the last exercise session (n = 5–6 in each age and group). Significant differences compared with P60^a^, P90^b^, P120^c^, or CTL group* (p < 0.05 by two-way ANOVA and Tukey post-test).

Tukey’s post-hoc test showed a transient alteration in the expression of cortical mTOR and hippocampal p70S6K and CREB and in the activation of hippocampal Akt, and ERK was noted during rats’ aging, particularly when comparing P90 and other ages (p < 0.05) (Fig. 4). When studied groups were compared (EX *vs* CTL), statistical analysis revealed that early physical exercise increased cortical mTOR expression at P60, but this protein was restored to control levels at P90 and P120 (p > 0.05) (Fig. 4).

## Discussion

It has been reported that individuals who were more physically active in early life have better cognitive and academic performance over their life course^1–3^. It is possible that early-life physical activity, similar to early-life education^18^, builds a neural reserve that has long-lasting benefits. To explore this issue, the present study investigated whether physical exercise in young rats, at a stage similar to childhood and adolescence in humans, induces long-lasting effects on cortical and hippocampal neurons. In our study, more neuronal cells were found in the cerebral cortex and hippocampus of exercised rats than in sedentary ones, except in hippocampal neurons at P90. Although the method we used did not allow cellular quantification by regions, our findings accord with previous studies^6,8,11,19,20^. For instance, an increase in the proliferation of hippocampal cells has been observed in exercised rats when compared with non-exercised rats^19^. A recent study by Victorino and collaborators^8^ noted a higher number of neuronal and non-neuronal cells in the cerebral cortex of adolescent rats submitted to exercise. Similar findings have shown a potent cellular proliferative effect of aerobic exercise on young rats’ brains in both the cortical and hippocampal structures^8,19^. Our data complement and extend these findings by showing that early exercise increases, and maintains elevated, the number of neurons in the cerebral cortex and hippocampus even during a sedentary period of rats. In other words, we observed that, unlike muscles^21^, early exercise-induced cellular effects did not return to normal levels after a long period of physical inactivity. However, it is important to point out that although the isotropic fractionator is an efficient method to identify small differences in cell count, we must consider that were analyzed neurogenic and non-neurogenic hippocampal areas together. It may have increased the noise in the sample and contributed to the reduction of significance in the comparison between groups at age P90.

Although exercise-induced proliferative effect in the hippocampal cells is largely accepted^6,19,20^, new cell formation in the postnatal cerebral cortex is still very controversial^22,23^. The cellular increase found in our study suggest that our results may be linked to two typical conditions of postnatal brain development, such as neurogenesis and neuronal death. In hippocampal formation, Kim *et al*.^19^ and Almeida *et al*.^20^ showed neurogenesis in dentate gyrus of rats exercised during postnatal brain development. In study of Bechara *et al*.^14^ is observed that the practice of exercise increased BDNF, a neurotrophic factor related with neuronal survival. These data affirm our suggestion on the increase of hippocampal cells. However, the increase of cells that find in the cortex over time (i.e., from P60 to P120) seems to be more related to cell survival, alterations in vessels and/or glial cells cortex^24–26^ because lack of evidence to support the cortical neurogenesis^27^. About this, in a research conducted by Noctor *et al*.^28^ was noted that cortical neurogenesis occurs only in the first week of postnatal life. In contrast, Bandeira *et al*.^29^, using the isotropic fractionator, observed a significant increase of cortical neurons in rats at P25 and P70 compared to animals at P15. Thus, to confirm the pathway that generated an increase in cortical cells found in our study, it is necessary to carry out research using specific cellular markers for neurogenesis and cell survival.

In our study, early exercise also increased the number of dendrites, total dendritic length, number of dendritic nodes and terminal ends of cortical neurons of layers III–V and hippocampal neurons of the CA1 over the life course of rats. Although the number of dendrites of hippocampal neurons was lower in the exercised animals in relation to the control animals at P60, no difference was observed in total number, nodes and dendritic endings. This shows that the neurons of the exercised animals cross an area and have a complexity similar to those of the control group and that hippocampal (CA1) and cortical (sensory and motor) regions are distinctly influenced by the practice of physical exercise. In brief, Sholl analysis conducted on cortical and hippocampal neurons showed that dendritic arborization was higher in exercised than in control rats. These data corroborate findings from previous studies which report a greater cortical and hippocampal dendritic structure and complexity in physically active rodents^11,30–32^. Since increased cell number and large dendritic branching induced by exercise are accompanied by facilitated LTP and cognitive improvement^6,32^, we hypothesized that early exercise-induced lasting morphological changes on cortical and hippocampal neurons may result in the development of more complex neural circuitry. This enhanced plasticity could be capable of improving lifelong brain functions and tolerating brain damages in later life. Indeed, early exercise has been shown to be positively associated with better cognitive and academic performance throughout life^1,2^, and this, in turn, has been linked with lower rates of cognitive impairment in older age^3,33,34^. Moreover, it has been found in population-based cohort studies involving over 1 million individuals that higher early life aerobic fitness is associated with lower risk for schizophrenia and schizophrenia-like disorders^35^, epilepsy and serious depression in adulthood^36,37^, and early-onset dementia and mild cognitive impairment later in life^38^. To better understand these early exercise-induced neuroprotective effects, in a previous study we evaluated whether juvenile exercise in rats (P21–P60) modifies their later seizure susceptibility induced by the pilocarpine model of epilepsy at P150^39^. The results showed that early exercise delayed the onset and reduced the intensity of pilocarpine-induced motor symptoms in midlife rats^39^. These findings corroborate population data showing that physical activity habits at early ages may interfere positively in later epilepsy development^38^ and support the hypothesis that juvenile exercise may build a neural reserve against neurological disorders. These benefits may be associated with the cellular and dendritic changes induced by early exercise.

These data are important because increase in the neuronal morphology and population induced by early exercise can have a significant impact on the brain structure and function during aging. It has been observed that physically active children have greater hippocampal volume that is associated to a better cognitive performance in mnemonic tests^40,41^. This beneficial effect of exercise has also been found in pre-adolescents, adolescents^42^, adults^7^ and elder^2,3^. In addition, scientific data have reported that a healthy lifestyle is capable of generating a brain reserve that may be useful during aging^4,18,34^, especially in degenerative and brain pathological processes, such as ischemia, dementia and Alzheimer’s and Parkinson’s diseases^4,43^. Together with our results, these data suggest that the practice of physical exercise during brain development can promote changes in the cortical and hippocampal structures of the brain that can be used throughout aging. However, we did not assess the cognitive status of the animals in our study. This limits our ability to assert the functionality of the neural reserve in our experimental model.

Nonetheless, how did these changes remain throughout of a sedentary period of rats after the exercise ended? These early exercise-induced lasting cellular and dendritic changes may be related to the developmental period during which the stimulus was performed. During the postnatal period of brain development, several morphological and functional changes occur in neural circuits^44,45^. When new neurons are produced, their dendrites branch to establish synapses between cells^46,47^. During this period, there is a cellular and synaptic process of elimination known as “pruning”, supposedly to remove inappropriate connections and maintain those that are suitable for the brain^48^. Neuronal and synaptic pruning is characteristic of the adolescent period^44^, a life stage extremely vulnerable to disruption and alteration^49^. Bearing in mind that the developing brain may be easily affected by physical activity^5^, we hypothesized that aerobic exercise undertaken during the adolescent period may have slowed down or even delayed the pruning process, thus resulting in lasting effects on neuronal morphology.

Here, we sought to determine whether the morphological changes induced by exercise are accompanied by lasting alterations in the brain expression of proteins linked to cell growth, proliferation and survival. To do this, we evaluated Akt, mTOR, p70S6K, ERK and CREB expression and activation (phosphorylated/total protein ratio) and BDNF levels in the cerebral cortex and hippocampal formation throughout of sedentary period of rats submitted to aerobic exercise during the adolescent period. It was observed that juvenile exercise increased hippocampal BDNF levels and cortical mTOR expression at P60, as previously described in the literature^7,8^. However, these effects were not maintained over the sedentary period. Levels and expression of these proteins were restored to control levels when evaluated at P90 and P120. This result was not expected, since higher BDNF levels in the perirhinal cortex of adolescent rats have been found 14 and 30 days after an exercise period^9^. Negative effects may appear in inappropriate conditions of physical stress, so we also examined whether the exercise-induced transient effect on BDNF and mTOR might have been influenced by an increase in stress hormones, such as ACTH and corticosterone. It is known that chronic stress can stimulate the HPA axis and suppress the release of BDNF in the brain^50,51^. Nevertheless, no significant change in cortical and hippocampal ACTH and corticosterone levels was detected in this study. Thus, we assumed that juvenile exercise-induced lasting cellular and dendritic effects are not associated with changes in brain levels of BDNF, Akt, mTOR, p70S6K, ERK and CREB, ACTH and corticosterone. However, it is possible that early exercise may have promoted long-lasting changes in the epigenome of BDNF and other molecules that are not expressed in protein levels until needed^52^.

In our study, a peak of hippocampal BDNF was observed in both groups at P90. It is known that BDNF levels are regulated for specific brain regions according to the developmental process^53^. For example, studies using long mRNA probes with nuclease protection assay indicate that the expression of BDNF in the prefrontal cortex of humans is highest in period of maturation of the prefrontal cortex^54^ and maintained throughout aging. In rats, peak of the cortical BDNF levels is observed at P20, being gradually reduced until reaching 50%. In the hippocampus, BDNF level increase gradually between P20 and P120, until reaching a plateau point^55,56^. This data differs from our results. About this, BDNF transcription is regulated by 8 different promoters which can yield different levels of expression depending on the promoter under study, and this can explain the difference across studies^57^. In our case we used the multiplex system to measure total BDNF protein, and the details of the BDNF detection are proprietary. However, the overall results seem to indicate that the peak of hippocampal BDNF at P90 shown by us coincides with the end of the neonatal period described by other studies^54–56,58^.

In conclusion, our results show that, despite short-term effects on growth and survival proteins, juvenile exercise induced lasting morphological changes in cortical and hippocampal neurons throughout of a sedentary period of rats. Further studies should be conducted to evaluate the early exercise-induced neural reserve in more challenging situations, such as chronic stress or brain injury. These findings have important implications for human health as they support the idea that exercising during youth results in sustained brain health and cognitive benefits^1,2,9^.

## Methods

### Exercise paradigm

**Figure 5:**
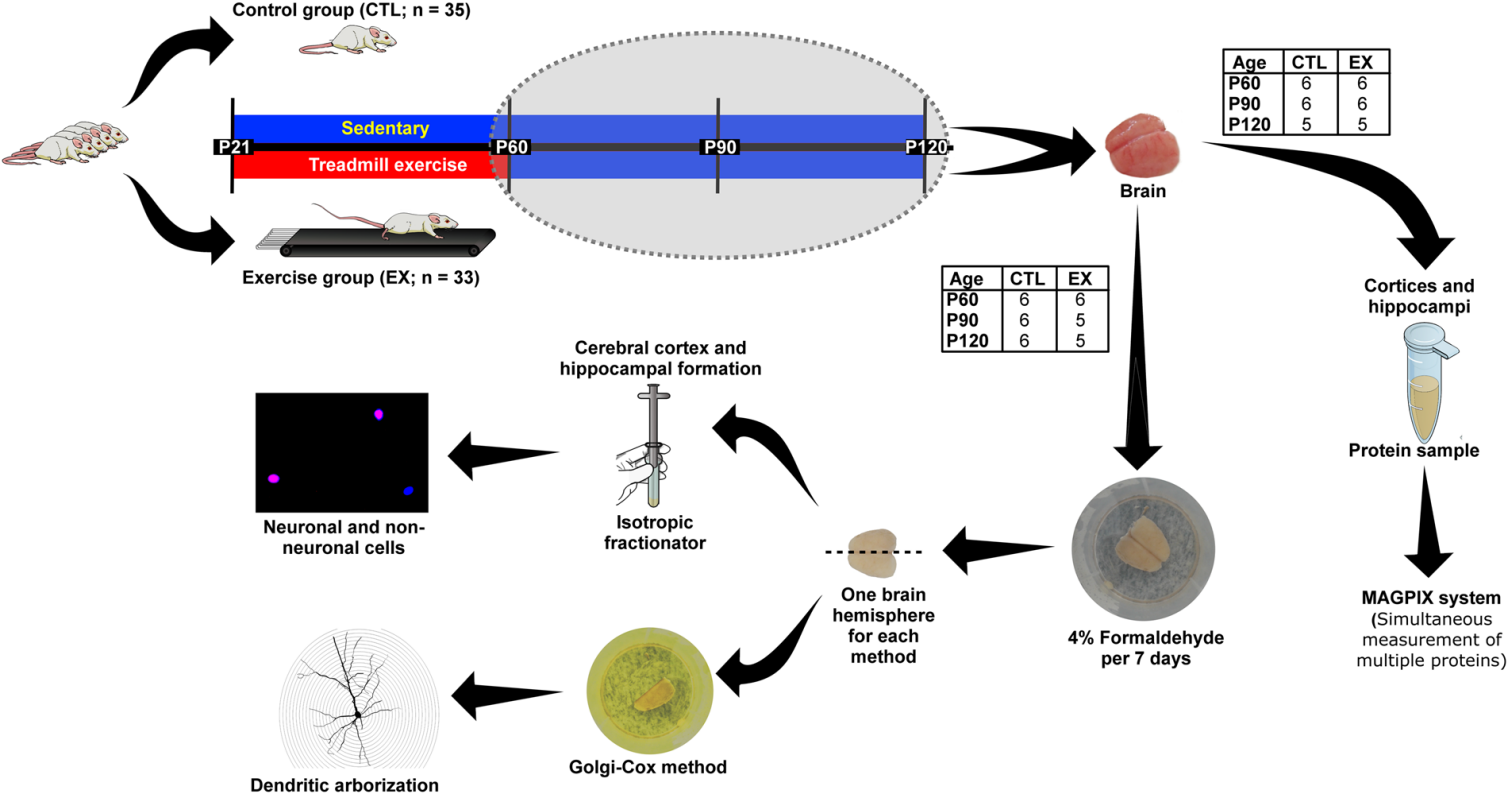
Experimental design. Male Wistar rats from the exercise (EX) group were submitted to an aerobic exercise protocol between the 21^st^ and 60^th^ postnatal days (P21–P60), while rats from the control (CTL) group were kept on a stopped treadmill for the same amount of time and circadian period. Subsequently, they were investigated at different life stages: 0 (P60), 30 (P90) and 60 (P120) days after the last exercise session. Their brains (n = 5–6 in each age and group) were removed and postfixed with paraformaldehyde to evaluate the absolute number of neuronal (in pink) and non-neuronal (in blue) cells using the isotropic fractionator method, and dendritic arborization was evaluated using the Golgi-Cox method (one brain hemisphere for each method). A different set of rats from the EX and CTL groups (n = 5–6 in each age and group) was used to investigate the expression of growth, proliferation and survival proteins (BDNF, Akt, mTOR, p70S6K, ERK and CREB) and stress hormones (ACTH and corticosterone) by simultaneous measurement of multiple proteins with the MAGPIX system.

Seventy-two male Wistar rats at postnatal day 21 (P21) were distributed into exercise (EX; n = 33) and control (CTL; n = 35) groups. The colony room was maintained at 21 ± 2 °C with a 12 h light/dark schedule (light: 7am to 7 pm), and *ad libitum* food and water was provided throughout the experiments. Rats from the EX group were submitted to an aerobic exercise program on treadmill (Columbus Instruments) from the 21^st^ to the 60^th^ postnatal days (P21–P60), as previously described by Gomes da Silva and collaborators^59^. Starting with a 3 min warm-up at 8 m/min. Both, running time and speed were progressively increased up to 18 m/min for 60 min, at ages P21 to P60. Rats from the CTL group were kept on a stopped treadmill under the same amount of time and same circadian periods as the exercise group. Afterwards, the absolute number of cells, neuronal branching and expression of growth, proliferation and survival proteins (BDNF, Akt, mTOR, p70S6K, ERK and CREB) and stress hormones (ACTH and corticosterone) in the cerebral cortex and hippocampal formation were investigated at different life stages: 0 (P60), 30 (P90) and 60 (P120) days after the last exercise session (Fig. 5). The rats were euthanized between 09:00 a.m. and 1:00 p.m. To maintain homogeneity of the results, mainly of the protein expression data, one animal at a time was euthanized from each group. All experimental protocols were approved by the Ethics Committees of the Hospital Israelita Albert Einstein (SGPP protocol #1920-13), Universidade Federal de São Paulo (CEUA #5067101115) and Universidade de Mogi das Cruzes (CEUA #003/2016). All efforts were also made to minimize animal suffering in accordance with the proposals of the International Ethical Guidelines for Biomedical Research^60^.

### Isotropic fractionator method

Isotropic fractionator method was used to analyze the he total number of neuronal and non-neuronal cells in the cerebral cortex and hippocampal formation^61^. This method replicates approximately all the outcomes produced using unbiased stereology techniques which validates it for determining the absolute number of neuronal and non-neuronal cells^62–64^. For this, one brain hemisphere from each rat (n = 5–6 in each group and age) was removed immediately after euthanasia and placed in a solution containing 4% paraformaldehyde in 0.1 M phosphate-buffered saline (pH 7.40) for one week. Subsequently, the cerebral cortex (all cortical tissue) and hippocampal formation (Cornus Ammon and dentate gyrus) were dissected by means of consistent anatomical landmarks. The cerebral cortex comprised all regions dorsolateral to the olfactory tract, excluding the hippocampal formation, and was dissected from each hemisphere by peeling it away from the striatum and other subcortical structures^65^. The assay was conducted in accordance to the method described by Herculano-Houzel and Lent^61^.

### Golgi-Cox method

Neuronal branching from the cerebral cortex (layers III–V) and hippocampus (Cornu Ammonis 1; CA1) were evaluated in stained sections using the Golgi-Cox method (n = 10 neurons/region per animal) (Bregma: −3.50 to −4.50 mm)^66^. CA1 hippocampal region was chosen because it is a region sensitive to plasticity and it acts effectively in receiving and sending information in the communication between the entorhinal cortex and hippocampal formation. In addition, the CA1 has a larger extension and, consequently, has a greater amount of pyramidal cells compared to the other hippocampal regions^65,67,68^. Therefore, we believe that the CA1 layer is well suited to observe effects of brain plasticity, particularly concerning fine neuronal structure as shown by the Golgi-Cox method. In brief, one brain hemisphere from each rat (n = 5–6 of both groups and ages) was removed immediately after decapitation and placed in a solution containing 4% formaldehyde in 0.1 M phosphate-buffered saline (pH 7.40) for one week. Brain hemispheres from studied groups were then submitted to the Golgi-Cox staining method^69^. The Golgi-Cox technique involves two sequential incubation processes. The first consists of immersing the tissue in a fixative solution over 14 days in a dark environment, and the second of keeping it in a solution of K_2_Cr_2_O_7_ (1%) for 24 hours. The fixative solution was obtained by mixing three different types of solutions, described below as A, B and C. Solution A was obtained by dissolving 10 g of HgCl_2_ in 200 milliliters of distilled water at a temperature of 80 °C for 15 to 20 minutes. Solutions B and C were respectively obtained by dissolving 10 g of K_2_Cr_2_O_7_ and 20 g of K_2_CrO_4_ in 200 and 600 milliliters of distilled water at room temperature. Finally, solutions A, B and C were mixed under slow shaking until homogenization. Tissue with the fixative solution must be kept at rest and protected from light, and after a minimum of 12 hours, the Golgi-Cox solution can be used for staining neurons.

After the incubation processes, brain hemispheres were sectioned coronally in a vibratome (mod. VT1000S, Leica) at 150 micrometers (μm) thickness. The sections were then assembled on gelatinized slides and dried at room temperature for approximately 3 hours. The dried slices were immersed in 28% ammonium hydroxide (NH_4_OH) and 15% Kodak Fixer respectively for 30 and 15 minutes. Subsequently, slices were washed in distilled water for a period of 5 minutes and dehydrated in a 50%, 75%, 95% and 100% alcohol incremental battery for a period of 3 minutes for each concentration. At the end of dehydration, the glass slide was diaphanized with xylol for 3 minutes and coverslipped with Entellan^®^ (Merk).

Pyramidal neurons from the regions of interest (cerebral cortex and hippocampal formation) were traced at 60x total magnification using Neurolucida Image Analysis System (Microbrightfiel, Bioscience), installed on a computer connected to an optical microscope with a 3D motorized platform (X, Y and Z axes; Zeiss, Imager M2). After neuron reconstruction, specific neuronal structures and dendritic complexity were analyzed using Neuroexplorer software (Microbrightfield, Bioscience). The total amount and length of dendrites, bifurcations (nodes) and dendritic endings (terminal ends) of each neuron were quantified. Quantitative data of dendritic complex arborization were obtained by Sholl analysis^70^. In this analysis, virtual concentric circumferences are positioned with their radius originating from the center of the soma of each neuron, starting with a radius of 10 μm. The subsequent circumferences had radii increased by 10μm each time. In this way, all intersections were quantified for each ring.

### Simultaneous measurement of multiple proteins with MAGPIX system

The MAGPIX system was used to evaluate cortical and hippocampal expression of BDNF, ACTH, corticosterone, Akt, mTOR, p70S6K, ERK and CREB. For this purpose, the cortices and hippocampi of rats from the exercise and control groups (n = 4–6 in each group and age) were removed immediately after decapitation and homogenized in 0.01 M Tris hydrochloride (pH 7.6) containing 5.8% of sodium chloride, 10% of glycerol, 1% of Nonidet P40 (NP-40), 0.4% of ethylenediamine tetraacetic acid and commercial kits of protease (M222-1 ml; Amresco) and phosphatase (Cat #B15001-A and B; Biotool) inhibitors. Animals from the EX group were euthanized 1 hour (P60), 30 (P90) and 60 (P120) days after the last exercise session. Animals from the CTL group were euthanized similar to the EX group (1 h after they stayed on a stopped treadmill at P60, P90, and P120). Samples were sonicated and stored at −80 °C. Kits were then used to evaluate the BDNF and ACTH (RPTMAG-86K; EMD Millipore), corticosterone (RSHMAG-69K; EMD Millipore), Akt total and phosphorylated (48-618MAG; EMD Millipore), ERK total and phosphorylated (48-619MAG; EMD Millipore), mTOR total (46-685AMAG; EMD Millipore) and phosphorylated (Ser2448) (46-686AMAG; EMD Millipore), p70S6K total (46-630MAG; EMD Millipore) and phosphorylated (Thr412) (46-629AMAG; EMD Millipore), and CREB total (46-632MAG; EMD Millipore) and phosphorylated (Ser133) (46-631AMAG; EMD Millipore), The assay was carried out according to the manufacturer’s specifications.

### Statistical analysis

The two-way ANOVA for repeated measures and Tukey post-hoc tests were used for the statistical analysis. The extreme value test (Grubbs) was used to remove outlier values. All values were considered significant when p < 0.05. Data are presented as mean and standard error of the mean (±SEM).

## Supplementary information

Supplementary data
