# Supplementary material for: Early exercise induces long-lasting morphological changes in cortical and hippocampal neurons throughout of a sedentary period of rats
Source: Sci Rep. 2019 Sep 23;9:13684. doi: 10.1038/s41598-019-50218-9 (PMC6757043; doi:10.1038/s41598-019-50218-9)
Supplement: Supplementary file 1 — Supplementary data [file 41598_2019_50218_MOESM1_ESM.docx]

**Supplementary information**

**Title of the manuscript:** Early exercise induces long-lasting morphological changes in cortical and hippocampal neurons throughout of a sedentary period of rats

**Author list:** Fernando Tadeu Serra, Andrea Dominguez Carvalho, Bruno Henrique Silva Araujo, Laila Brito Torres, Fabrizio dos Santos Cardoso, Jéssica Salles Henrique, Eduardo Varejão Díaz Placencia, Roberto Lent, Fernando Gomez-Pinilla, Ricardo Mario Arida, Sérgio Gomes da Silva.

**Supplementary Table 1.** Two-way ANOVA results for the absolute number of neuronal and non-neuronal cells

| **Variable** | **Statistical values** | | | | | |
| --- | --- | --- | --- | --- | --- | --- |
|  | **Age** | | **Group** | | **Interaction** | |
| Cortical neurons | F_(2, 27)_ = | 16.3, p < 0.001* | F_(1, 27)_ = | 138, p < 0.001* | F_(2, 27)_ = | 2.46, p = 0.105 |
| Cortical non-neuronal cells | F_(2, 27)_ = | 47,4, p < 0.001* | F_(1, 27)_ = | 60.4, p < 0.001* | F_(2, 27)_ = | 1.79, p = 0.186 |
| Hippocampal neuronal | F_(2, 26)_ = | 1.00, p = 0.381 | F_(1, 26)_ = | 66.6, p < 0.001* | F_(2, 26)_ = | 2.69, p = 0.086 |
| Hippocampal non-neuronal cells | F_(2, 28)_ = | 10.6, p < 0.001* | F_(1, 26)_ = | 31.9, p < 0.001* | F_(2, 28)_ = | 7.24, p = 0.003* |

**Supplementary Table 2.** Two-way ANOVA results for the number of dendrites, dendritic nodes and ends, and total dendritic length.

| **Variable** | | **Statistical values** | | | | | | | | |
| --- | --- | --- | --- | --- | --- | --- | --- | --- | --- | --- |
|  |  | **Age** | | | **Group** | | | **Interaction** | | |
| **cerebral cortex (III - V layers)** | Number of dendrites | F_(2, 334)_=22.1, | p < | 0.001* | F_(1, 334)_=90.4, | p < | 0.001* | F_(2, 334)_=10.1, | p < | 0.001* |
|  | Total dendritic length | F_(2, 334)_=16.4, | p < | 0.001* | F_(1, 334)_=324, | p < | 0.001* | F_(2, 334)_=25.9, | p < | 0.001* |
|  | Number of dendritic nodes | F_(2, 334)_=48.9, | p < | 0.001* | F_(1, 334)_=237, | p < | 0.001* | F_(2, 334)_=0.165, | p = | 0, 848 |
|  | Number of dendritic ends | F_(2, 334)_=51.9, | p < | 0.001* | F_(1, 334)_=267, | p < | 0.001* | F_(2, 334)_=0.321, | p = | 0.726 |
| **Hippocampus (CA1)** | Number of dendrites | F_(2, 324)_=47.6, | p < | 0.001* | F_(1, 324)_=0.265, | p = | 0.607 | F_(2, 324)_=33.6, | p < | 0.001* |
|  | Total dendritic length | F_(2, 324)_=167, | p < | 0.001* | F_(1, 324)_=44.8, | p < | 0.001* | F_(2, 324)_=9.15, | p < | 0.001* |
|  | Number of dendritic nodes | F_(2, 324)_=227, | p < | 0.001* | F_(1, 324)_=92.8, | p < | 0.001* | F_(2, 324)_=42.8, | p < | 0.001* |
|  | Number of dendritic ends | F_(2, 234)_=246, | p < | 0.001* | F_(1, 324)_=92.2, | p < | 0.001* | F_(2, 324)_=54.8, | p < | 0.001* |

**Supplementary Table 3.** Two-way ANOVA results for cortical and hippocampal levels of BDNF, ACTH and corticosterone.

| **Variable** | | **Statistical values** | | | | | | | | |
| --- | --- | --- | --- | --- | --- | --- | --- | --- | --- | --- |
|  |  | **Age** | | | **Group** | | | **Interaction** | | |
| **Cortices** | BDNF | F_(2, 22)_ = 0.113, | p = | 0.894 | F_(1, 22)_ = 0.786, | p = | 0.385 | F_(2, 23)_ = 0.937, | p = | 0.407 |
|  | ACTH | F_(2, 22)_ = 0.072, | p = | 0.930 | F_(2, 23)_ = 6.55, | p = | 0.018* | F_(2, 22)_ = 1.86, | p = | 0.179 |
|  | Corticosterone | F_(2, 22)_ = 1.95, | p = | 0.166 | F_(1, 23)_ = 0.087, | p = | 0.771 | F_(2, 23)_ = 0.460, | p = | 0.637 |
| **Hippocampi** | BDNF | F_(2, 29)_ = 119, | p < | 0.001* | F_(1, 29)_ = 0.516, | p = | 0.479 | F_(2, 29)_ = 10.4, | p < | 0.001* |
|  | ACTH | F_(2, 27)_ = 0.833, | p = | 0.446 | F_(1, 27)_ = 0.023, | p = | 0.882 | F_(2, 27)_ = 0.639, | p = | 0.536 |
|  | Corticosterone | F_(2, 27)_ = 3.02, | p = | 0.065 | F_(1, 27)_ = 3.15, | p = | 0.087 | F_(2, 27)_ = 1.90, | p = | 0.169 |

**Supplementary Table 4.** Two-way ANOVA results for cortical and hippocampal expression of Akt, mTOR, p70S6K, ERK and CREB.

| **Variable** | | **Statistical values** | | | | | | | | |
| --- | --- | --- | --- | --- | --- | --- | --- | --- | --- | --- |
|  |  | **Age** | | | **Group** | | | **Interaction** | | |
| **Cortices** | Akt | F_(2, 25)_ = 4.23, | p = | 0.026 | F_(1, 25)_ = 0.179, | p = | 0.895 | F_(2, 25)_ = 0.928, | p = | 0.409 |
|  | mTOR | F_(2, 26)_ = 5.73, | p = | 0.009* | F_(1, 26)_ = 4.99, | p = | 0.034* | F_(2, 26)_ = 3.15, | p = | 0.059 |
|  | p70S6K | F_(2, 26)_ = 1.10, | p = | 0.349 | F_(1, 26)_ = 0.132, | p = | 0.719 | F_(2, 26)_ = 0.017, | p = | 0.983 |
|  | ERK | F_(2, 24)_ = 0.145, | p = | 0.866 | F_(1, 24)_ = 1.06, | p = | 0.314 | F_(2, 24)_ = 2.77, | p = | 0.083 |
|  | CREB | F_(2, 24)_ = 1.26, | p = | 0.302 | F_(1, 24)_ = 4.03, | p = | 0.560 | F_(2, 24)_ = 1.43, | p = | 0.258 |
| **Hippocampi** | Akt | F_(2, 25)_ = 1.27, | p = | 0.299 | F_(1, 25)_ = 0.547, | p = | 0.467 | F_(2, 26)_ = 1.89, | p = | 0.172 |
|  | mTOR | F_(2, 26)_ = 0.839, | p = | 0.443 | F_(1, 26)_ = 0.931, | p = | 0.343 | F_(2, 26)_ = 1.87, | p = | 0.173 |
|  | p70S6K | F_(2, 23)_ = 117, | p < | 0.001* | F_(2, 23)_ = 0.266, | p = | 0.611 | F_(2, 23)_ = 1.66, | p = | 0.212 |
|  | ERK | F_(2, 24)_ = 0.293, | p = | 0.749 | F_(1, 24)_ = 3.26, | p = | 0.084 | F_(2, 24)_ = 0.380, | p = | 0.688 |
|  | CREB | F_(2, 23)_ = 11.9, | p < | 0.001* | F_(2, 23)_ = 2.46, | p = | 0.131 | F_(2, 23)_ = 1.74, | p = | 0.197 |

**Supplementary Table 5.** Two-way ANOVA results for cortical and hippocampal activation (phosphorylated/total protein) of Akt, mTOR, p70S6K, ERK and CREB.

| **Variable** | | **Statistical values** | | | | | | | | | | | |
| --- | --- | --- | --- | --- | --- | --- | --- | --- | --- | --- | --- | --- | --- |
|  |  | Age | | | | Group | | | | Interaction | | | |
| **Cortices** | Akt (p/t) | F_(2, 25)_ = | 0.598, | p = | 0.558 | F_(1, 25)_ = | 2.16, | p = | 0.155 | F_(2, 25)_ = | 0.691, | p = | 0.510 |
|  | mTOR (p/t) | F_(2, 26)_ = | 0.418, | p = | 0.663 | F_(1, 26)_ = | 0.003, | p = | 0.955 | F_(2, 26)_ = | 0.336, | p = | 0.717 |
|  | p70S6K (p/t) | F_(2, 27)_ = | 4.47, | p = | 0.022* | F_(1, 27)_ = | 1.45, | p = | 0.239 | F_(2, 27)_ = | 0.406, | p = | 0.670 |
|  | ERK (p/t) | F_(2, 24)_ = | 1.03, | p = | 0.371 | F_(1, 24)_ = | 0.652, | p = | 0.427 | F_(2, 24)_ = | 0.468, | p = | 0.621 |
|  | CREB (p/t) | F_(2, 23)_ = | 2.63, | p = | 0.094 | F_(1, 23)_ = | 1.22, | p = | 0.281 | F_(2, 23)_ = | 0.294, | p = | 0.679 |
| **Hippocampi** | Akt (p/t) | F_(2, 25)_ = | 26.3, | p < | 0.001* | F_(1, 25)_ = | 0.751, | p = | 0.394 | F_(2, 25)_ = | 0.823, | p = | 0.451 |
|  | mTOR (p/t) | F_(2, 27)_ = | 0.511, | p = | 0.950 | F_(1, 27)_ = | 6.05, | p = | 0.021* | F_(2, 27)_ = | 1.45, | p = | 0.253 |
|  | p70S6K (p/t) | F_(2, 23)_ = | 2.67, | p = | 0.090 | F_(1, 23)_ = | 3.52, | p = | 0.074 | F_(2, 23)_ = | 3.08, | p = | 0.065 |
|  | ERK (p/t) | F_(2, 24)_ = | 21.2, | p < | 0.001* | F_(1, 24)_ = | 0.106, | p = | 0.747 | F_(2, 24)_ = | 3.87, | p = | 0.035* |
|  | CREB (p/t) | F_(2, 24)_ = | 0.890, | p = | 0.424 | F_(1, 24)_ = | 1.77, | p = | 0.196 | F_(2, 24)_ = | 0.701, | p = | 0.506 |

p/t = phospho/total protein ratio.
